# Feasibility of alcohol screening and brief intervention in primary health care in Kazakhstan: study protocol of a pilot cluster randomised trial

**DOI:** 10.1186/s40814-019-0547-x

**Published:** 2020-01-09

**Authors:** Bernd Schulte, Amy O’Donnell, Harald Lahusen, Christina Lindemann, Mariya Prilutskaya, Oleg Yussopov, Zhanar Kaliyeva, Marcus-Sebastian Martens, Uwe Verthein

**Affiliations:** 1 Centre of Interdisciplinary Addiction Research of Hamburg University, Department of Psychiatry, University Medical Centre Hamburg-Eppendorf, Martinistr. 52, 20246 Hamburg, Germany; 2 Population Health Sciences Institute, Newcastle University, Newcastle upon Tyne, UK; 3 Pavlodar Branch of Semey State Medical University, Pavlodar, Kazakhstan; 4 Monitoring Center on Alcohol and Drugs, Pavlodar, Kazakhstan; 5 Sanjar Dzhafarovich Asfendiyarov Kazakh National Medical University, Almaty, Kazakhstan

**Keywords:** Alcohol, Screening, Brief intervention, Primary health care, Implementation processes

## Abstract

**Background:**

Identifying and addressing heavy drinking represents a major public health priority worldwide. Whilst the majority of alcohol screening and brief intervention (ASBI) research has been conducted in western, high-income countries, evidence is growing that ASBI can also impact positively on heavy drinkers in low- and middle-income country populations. This mixed methods study aims to assess the feasibility of conducting a fully randomised controlled trial of the effectiveness of ASBI in primary care in Kazakhstan and explore the feasibility and acceptability of implementing ASBI in this setting from patients’ and physicians’ perspectives.

**Methods:**

Six primary health care units in the region of Pavlodar will be cluster randomised to either an intervention (WHO manualised 5 min alcohol brief intervention plus alcohol leaflet) or control group (simple feedback plus alcohol leaflet). Primary feasibility measures will be rates of participation at baseline and retention of eligible patients at the 3-month follow-up point. Patient/physician questionnaires and physician focus groups will assess additional dimensions of feasibility, as well as acceptability, according to the RE-AIM framework: Reach (rates of eligible patients screened/received advice); Effectiveness (change in AUDIT-C score); Adoption (rate/representativeness of participating physicians); Implementation (quality of ASBI/barriers and facilitators to delivery); and Maintenance (potential sustainability of intervention).

**Discussion:**

This is the first trial of the feasibility and acceptability of ASBI in Kazakhstan. As the planning and assessment of implementation determinants is based on the RE-AIM framework, the project outcomes will be relevant for the future development, tailoring and implementation of ASBI in Kazakhstan.

**Trial registration:**

DRKS, DRKS00015882, Registered 17 December 2018.

## Background

Alcohol is a leading global risk factor for premature death and disability, and causally related to over 60 different medical conditions, including liver cirrhosis, cancer and cardiovascular disease [1–4]. Alcohol harm contributes to health inequalities, with a larger impact on younger age groups, mainly due to the increased risk of injuries, and higher alcohol attributable mortality rates for men compared to women [2, 5, 6]. Epidemiological data also confirm the greater alcohol-related disease burden experienced by socio-economically deprived and marginalised people [2, 7, 8]. Importantly, alcohol is a cause of significant harm to others, resulting in negative social and economic consequences, which extend beyond the individual drinker to their families, local communities and society as a whole [9–15]. As such, identifying and addressing heavy drinking represents a major public health priority worldwide [16, 17].

Globally, alcohol dependence is the most common substance use disorder; however, the prevalence of alcohol harm varies considerably across different populations [1]. In Central Asia, Kazakhstan displays particularly high levels of consumption; 7.7 L of alcohol per capita compared to 2.7 in Uzbekistan for example [18]. However, it remains challenging to determine the extent of excessive drinking in Kazakhstan due to lack of reliable data. Official statistics for 2010 suggest that 1.43% of the Kazakh population were drinking at dependent levels [19]. Yet, the World Health Organization (WHO) estimate the rate to be almost four times higher (5.2%) for the same year [20]. As in other former Soviet states, high levels of unrecorded alcohol production potentially contribute to this discrepancy. Within Kazakhstan itself, rates of alcohol consumption also vary, with the highest levels found in the Northern, Eastern and Central regions. In the Pavlodar region for example, the official prevalence of alcohol dependence in 2010 was about twice as high as that found nationally (2.3% vs. 1.4%) [19]. Whilst this difference has declined in recent years (0.75% vs. 0.61% in 2017) [21], Pavlodar continues to experience substantial alcohol-related harms, with higher rates of severe alcohol intoxications and mortality due to alcohol intoxication than the rest of the country. Compared to Kazakhstan as a whole, Pavlodar also reports a higher prevalence of hypertensive heart disease (six times higher), cardiac ischemia (23% higher), and trauma (61% higher). The mortality rate due to trauma, intoxication and accidents was found to be 30% higher in Pavlodar compared to the national average in 2016, with the number of deaths per capita caused by circulatory system diseases, 20% higher [22].

Primary health care (PHC) provides an ideal context for the early detection and secondary prevention of alcohol-related problems, due to its high contact exposure to the population [23] and the frequency with which excessive drinkers present to clinicians [24]. There is a particularly robust evidence for the delivery of alcohol screening and brief interventions (ASBI) in PHC, where patients tend to present with less acute symptoms, return regularly for follow-up appointments [25] and often build long-term relationships with their health care provider [26]. ASBI comprises two key elements. First, screening a patient to help identify those individuals drinking in a potentially hazardous or harmful way, with consistently good performance reported for both the Alcohol Use Disorder Identification Test (AUDIT) and its shorter consumption-focussed version (AUDIT-C) [27]. AUDIT was the first screening tool designed specifically to detect hazardous and harmful drinking in both primary and secondary care. Developed by the WHO AUDIT has ten questions that consider drinking frequency and intensity (binge drinking), together with experience of alcohol-related problems and dependence. At a score of eight or more out of a possible 40, its ability to detect genuine excessive drinkers (sensitivity) and to exclude false cases (specificity) is 92% and 94%, respectively. Thus, AUDIT is a highly accurate tool which has been validated in a large number of countries with consistently strong psychometric performance. It is now regarded as the ‘gold standard’ screening tool to detect hazardous and harmful drinking in primary care patients. A meta-analysis confirmed that the AUDIT-C is almost as accurate as the full AUDIT for detecting unhealthy alcohol use in adults [28]. Second, delivery of a brief behavioural intervention, designed to promote awareness of the negative effects of drinking and to motivate change [29, 30]. Across a series of systematic reviews, it has been consistently reported that brief alcohol interventions result in reduced weekly alcohol consumption [31] alcohol-related problems [32], healthcare utilisation [33] and mortality outcomes [34].

Despite this evidence, which is endorsed by the WHO [35] and embedded in clinical guidelines in Europe, Australasia and the USA [36–39], delivery of brief alcohol advice across global health systems remains low [40–42]. Moreover, even in countries where ASBI initiatives have been implemented, there has been limited evaluation of either their impact on overall delivery rates, particularly over the longer term, or to understand the mechanisms of change by which such improvements have been achieved. As a result, little is known about how and when effective ASBI interventions are implemented successfully in routine health care. In order to bridge this evidence to practice translation gap, a better understanding of the processes influencing how such health innovations and interventions are both taken up and sustained in practice is key. Further, whilst the majority of research has been conducted in western, developed countries, there is a growing body of evidence which suggests that ASBI can also impact positively on heavy drinkers in low- and middle-income country populations [43–45]. However, given the challenges experienced to date in achieving widespread implementation of alcohol interventions in countries where such preventative measures are already more established [46, 47], efforts to extend ASBI into novel settings must consider potential barriers and facilitators to effective adoption from the outset.

Standardised alcohol screening and brief interventions are not regularly implemented in PHC settings in Kazakhstan at present. Patients are asked about their alcohol consumption at health checks and receive advice as appropriate to help reduce their drinking. Where a potential alcohol use disorder is suspected, patients can be referred to specialised units for treatment. However, validated alcohol screening tools are not currently used, and the alcohol advice provided is not based on evidence-based guidelines. This protocol describes the rationale, methods and analysis plan for a pilot cluster randomised trial with embedded qualitative process evaluation that will explore the feasibility, acceptability and potential impact of implementing evidence-based ASBI in routine primary health care in Kazakhstan.

## Methods/design

### Aim

The primary aim of this study is to assess the feasibility of a proposed fully cluster randomised controlled trial of the effectiveness of ASBI in primary health care in Kazakhstan. The secondary aim is to explore the feasibility and acceptability of implementing ASBI in this setting from the perspective of patients and physicians.

Specific objectives are as follows:

1. To conduct a two-arm pilot feasibility cluster randomised controlled trial (cRCT) (with randomization at the level of PHCU) to assess the feasibility of a future definitive cRCT of ASBI in primary care in Kazakhstan;
2. To explore the feasibility and acceptability of ASBI and trial processes to physicians and patients;
3. To identify key determinants of ASBI implementation from the perspective of physicians and patients to identify specific facilitators and barriers to ASBI delivery in PHC;
4. To estimate the parameters for the design of a definitive cRCT of ASBI in primary care in Kazahkstan, including rates of eligibility, consent, participation and retention.

### Design

This pilot trial will use a two-arm cluster randomised design with embedded qualitative process evaluation to explore feasibility and acceptability in six PHC units (PHCU) in Kazakhstan. Stratified randomization with computer-generated random numbers will be used to allocate three PHCUs to the intervention and three PHCUs to the control group, with stratification based on an initial baseline assessment of the mean number of patient visits per PHCU per day.

#### Study duration

**Fig. 1:**
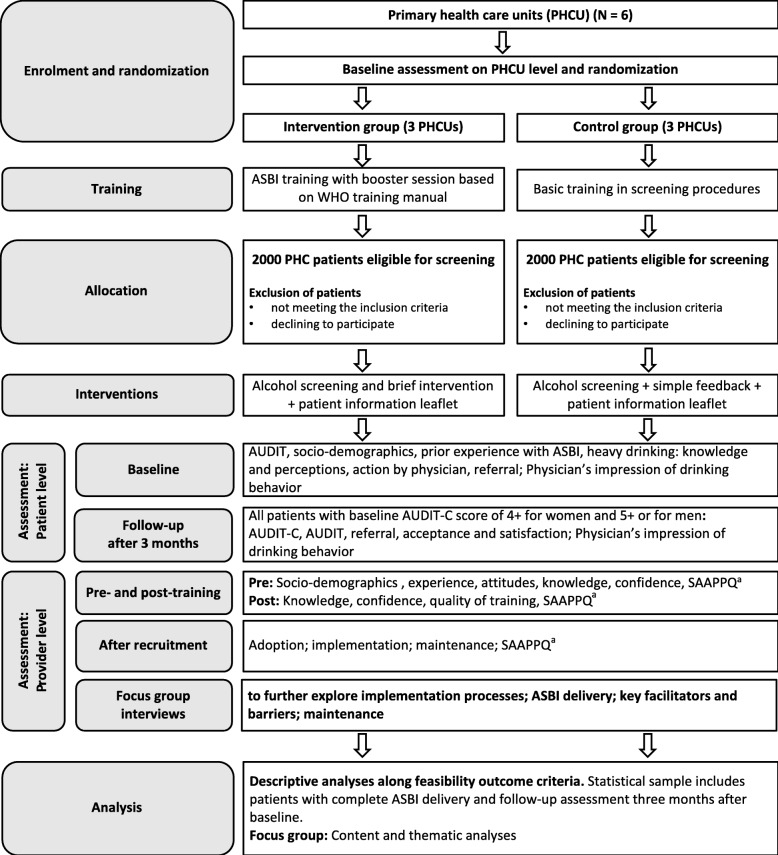
Consort flow-chart including cluster-randomised trial design. ^a^Shortened Alcohol and Alcohol Problems Perception Questionnaire

The study started in August 2018. The anticipated study duration is 15 months in total and expected to end in November 2019. This includes a 2-month site preparation phase following ethical approval from the Medical University of Almaty, 6-month recruitment period and follow-up assessments for each patient conducted 3 months after the patient was recruited (baseline). Introduction of the study and provider training sessions were conducted in April and May 2019. Patient recruitment started in April 2019. Figure 1 describes the study process in detail.

### Setting

The study will be carried out at six PHCUs in the Kazakh region of Pavlodar. All five PHCUs located in the city of Pavlodar and one PHCU the city of Aksu (region of Pavlodar), located about 40 km south of the city of Pavlodar. The list of participating PHCUs is provided in the trial register at www.drks.de.

The risk of contamination between intervention and control group is low, as every PHCU has its own catchment area and patients are only allowed to change their PHCU once a year between October and November. Furthermore, all participating physicians will be instructed to inquire whether a patient has already undergone screening in the same or any other PHCU, in order to prevent multiple screening and study contamination.

### Characteristics of participants

As a secondary preventative measure, an objective of ASBI is the early identification of individuals with hazardous or harmful alcohol use, followed by the delivery of a brief behavioural intervention to those in need of support to prevent the development of alcohol-related problems, including dependent drinking, in the longer term. This target group, by definition, is usually not seeking treatment for existing alcohol problems. However, individuals with hazardous or harmful drinking are likely to visit PHCUs for consultation on other health issues, which may be both related and/or unrelated to their drinking. Therefore, all patients with an appointment in one of the participating PHCUs are eligible for recruitment. Specific inclusion criteria are that participants must be aged between 18 and 69 years, able to follow the study procedures and have provided written informed consent. Patients with diagnosed lifetime alcohol dependency according to ICD-10 criteria will be excluded.

### Study processes

#### Recruitment

In both study arms, physicians will recruit patients presenting at routine care appointments. Patients potentially fulfilling the inclusion criteria will be informed about the study purpose and asked to take part. Each eligible patient who provides informed consent for study participation will be screened by a PHC physician for hazardous or harmful alcohol use using the three question Alcohol Use Disorder Identification Test – Consumption questionnaire (AUDIT-C) [48]. A show-card featuring the AUDIT-C questions and response options as well as illustrations of standard drink examples will be used as a visual aid for the screened patients. Additionally, all patients will be asked the remaining seven questions of the full AUDIT questionnaire in order to analyse correlations between AUDIT-C and full AUDIT scores [49]. The target population of the study consists of all PHC patients with risky, hazardous or harmful drinking, defined by an AUDIT-C score of four points or more for women and five points or more or for men. A full AUDIT score of 20 points or higher indicates a potential severe alcohol use disorder (AUD). If this is indicated and with the respective patient’s consent, these patients will be referred to a psychiatrist or narcologist for diagnostic assessment and treatment.

#### Intervention arm

In the intervention arm, patients with an AUDIT-C score of three or lower for females and four or lower for males will receive short verbal feedback based on their alcohol consumption and be given a patient information leaflet reinforcing the benefits of low-risk alcohol use. Patients with an AUDIT-C score of four or more for women and five or more or for men will receive a brief (5 min) face-to-face alcohol intervention delivered by a trained PHC physician, plus a patient information leaflet with recommendations on low-risk alcohol use. The intervention will be informed by motivational interviewing principles and structured around the FRAMES model (**F**eedback—provide feedback on the patient’s risk for harm; **R**esponsibility—the individual is responsible for change; **A**dvice—advise reduction or give explicit direction to change; **M**enu—provide a variety of options for change; **E**mpathy—take a warm, reflective and understanding approach; and **S**elf-efficacy—encourage optimism about changing behaviour) [50]. The advice will include extended personalised feedback aimed at increasing patient awareness about their drinking habits and related consequences and to enhance self-efficacy to change their drinking behaviour. In each PHCU, physicians will be provided with a 3-h training and a 1-h-booster session based on the WHO Europe ASBI training package, which has been previously adopted in Russia [51], and is comparable in content and length to the approach found to be effective in the European ODHIN trial [52]. Training will be delivered by local research staff experienced in using Motivational Interviewing techniques and in providing training to health professionals. Interactive activities and role play will be used during training sessions to build physician’s knowledge of/attitudes towards alcohol and alcohol-related harm, improve their understanding of key ASBI principles and components and boost physicians’ skills/capacity to implement ASBI in routine practice. A questionnaire will be administered to participants before and after training sessions to assess changes in knowledge, attitudes and self-rated work skills in relation in delivering ASBI in routine practice. Participants will also be asked to rate their satisfaction with the quality of training.

#### Control arm

PHC physicians in the control arm will be introduced to the study purpose, trained in the screening procedures with AUDIT-C and AUDIT including a respective booster session. All patients in the control arm will receive simple feedback, including information about their individual AUDIT-C/AUDIT score and the associated alcohol risk level, as well as a patient leaflet with recommendations for low-risk alcohol use to ensure a minimal standardised intervention.

### Data collection

#### Pre- and post-training

Provider experience, alcohol-related skills and attitudes to alcohol will be assessed prior to baseline using the Shortened Alcohol and Alcohol Problems Perception Questionnaire (SAAPPQ) [53]. Assessment of providers’ training needs will be based on the WHO training evaluation questionnaire which assesses, amongst other areas, work and training experience, knowledge of alcohol-related issues, and confidence in dealing with alcohol-related situations [51]. All physicians who decline to participate in the study will be asked to provide their work and training experience, as well as their reasons for not taking part.

#### Baseline assessments

Patient measures at baseline will be collected by case-report forms (CRF) and include socio-demographic characteristics (age, gender, ethnicity); knowledge of alcohol as an attributable factor for cancer, hypertension and liver disease; alcohol use screening score (AUDIT-C, AUDIT); and physician’s impression of drinking behaviour. Patient measures at follow-up (3 months post-baseline) will include alcohol use screening score (AUDIT-C); physician’s impression of drinking behaviour; patients’ view on the interventions received; patients’ acceptance of low-risk drinking recommendations; and patients’ view on relevance of the intervention (i.e. importance of information provided). In order to assess intervention reach, the socio-demographic characteristics of non-participating patients will also be documented.

#### Follow-up assessments

At the end of the recruitment period, participating physicians will be asked to provide their views on the implementation and potential maintenance of ASBI using structured questionnaires which will be administered by local research staff. The outcomes will inform qualitative focus groups (Table 1), which willl last approximately 60 to 90 min and will be moderated by an experienced researcher from the national study team. A semi-structured topic guide will be developed to focus discussions, drawing on key themes emerging from the structured questionnaire findings and the three major areas of interest—quality and consistency of ASBI delivered, barriers/facilitators to implementation of ASBI in PHC and the long-term sustainability of ASBI in this setting.

**Table 1:** Key outcome measures including domains of the RE-AIM framework [54]

Dimension	Outcome measures	Assessment tool and evaluation method
Feasibility	- *Rate of eligibility*: percentage of eligible participants recruited	Case-report form; descriptive statistics
	- *Participation and retention at 3 months follow-up*: percentage of enrolled participants retained at 3 months	
Reach	- *Proportion of patients screened at baseline* Numerator: Total number of completed screens Denominator: Total number of all patients eligible for screening	
	- *Proportion of consulting patients intervened at baseline* Numerator: Total number of screen-positive patients receiving advice Denominator: Total number of patients screened positive	
Effectiveness	- *Change in AUDIT-C* score between baseline and follow-up amongst patients with risky, hazardous or harmful drinking	
Adoption	- *Proportion of staff invited that participated* Numerator: Total number of participating physicians Denominator: Total number of primary care physicians invited	
	- *Description and representativeness of staff who delivered* the screening and advice (PHC provider level)	
Implementation	- *Quality and consistency of the delivery of ASBI* given (PHC provider level; patient level) measured by quantitative interviews, provider focus groups	Provider questionnaire; provider focus groups; descriptive statistics and qualitative data analyses (content and narrative analyses)
	- *Implementation determinants* (barriers, facilitators) of ASBI delivery (PHC provider, Patient level) collected by quantitative interviews, provider focus groups	
	- *Cost of intervention*: Average time spent in minutes per patient for screening and for brief intervention.	
Maintenance	- *Potential extent to which the intervention package becomes institutionalised* (PHC provider level; patient level) collected by quantitative interviews, provider focus groups	

#### Study outcome measures

The key outcome measures to assess feasibility will be the percentage of eligible patients recruited to the study at baseline and the retention rate of eligible patients at 3-month follow-up. Additional outcome measures to assess the feasibility and acceptability of implementing ASBI in primary care in Kazakhstan will be based on Glasgow’s RE-AIM framework for evaluating public health interventions [54]. RE-AIM consists of five dimensions (**R**each, **E**ffectiveness, **A**doption, **I**mplementation and **M**aintenance), which can be measured at the individual (patient/provider) or organisational (clinic/practice) level to help to determine the potential impact of an intervention.

Outcome measures relating to feasibility and each domain of the RE-AIM framework are detailed in Table 1.

#### Data management processes

Trial data will be collected using CRFs completed by the PHC physicians. Physicians will keep a code list of all patients who have scored AUDIT-C positive (4+ for females and 5+ for males) to ensure the anonymised matching of baseline and follow-up data. Trained local research staff will collect the anonymised data from the PHCUs and enter the data in a trial-specific database to monitor CRF delivery on a bi-weekly basis. Monitoring and query management (regular descriptive queries on central database to identify errors, outliers, missing data and any irregularities) will be carried out by national study coordinators. Original records containing any personal identifiable patient data will remain in the PHCUs.

Only physicians that provide written informed consent will be eligible to participate in the focus group interviews. Discussions will be audio-recorded, transcribed in full, with any potentially identifying details removed by the transcriber. Electronic recordings will be stored on password-protected computers for analysis and will be permanently deleted once the transcription process is completed. Data will be anonymised prior to analysis and write-up to ensure confidentiality and anonymity, i.e. participants will be identified by alpha-numeric code as opposed to a name. An anonymisation log (table) of all identifiers will be created to ensure consistency and accuracy, which will be stored separately to the interview data, and only accessible to members of the research team.

#### Data monitoring

External trained research staff will closely monitor the progress of the trial and ensure that the trial is conducted and recorded in accordance with the study protocol via bi-weekly site visits. PHC physicians are responsible for completion of the data sets. All PHC physicians will be briefed on the data documentation protocol in the training sessions. During the study, patient CRFs will be monitored continuously and checked for completeness, implausible values and correctness. If needed, corrections or additions will be made.

### Statistical analysis

Descriptive statistics will be used to evaluate quantitative outcome measures (e.g. rates of eligibility and retention). Given an anticipated drop-out rate to follow-up of 50%, statistical analyses on ASBI effectiveness will be based on the per protocol principle (PP). Due to the pilot character of the trial, a sample size calculation will not be made. However, on the basis of previous epidemiological data [55] we assume that 4000 PHC patients will need to be screened to result in a sample size of 400 patients with an AUDIT-C score of 4 or higher for women and 5 or higher for men. We anticipate a loss-to-follow-up-rate 3 months after screening of 50%, resulting in a sample size of 200 patients (i.e. 100 per group) for data analyses. By considering a moderate intra-class coefficient (ICC between 0.05 and 0.15) between the PHC units, this sample size will be sufficient to provide statistically significant results with a medium effect size (d between 0.4 and 0.6) for the analyses of the primary and secondary effectiveness endpoints. Paired *t* tests will be used for pre-post-comparison of drinking outcomes based on AUDIT-C score between baseline and follow-up within the groups. The qualitative data on the implementation processes and potential future maintenance will be summarized for a narrative analysis.

### Qualitative analysis and data integration

Content analysis will be used to explore and analyse the qualitative data gathered in the structured questionnaires and provider focus groups. Qualitative content analysis represents a systematic coding and categorisation approach that can be used to identify trends and patterns within large quantities of textual data [56] and has frequently been employed in applied health research. In this study, qualitative data from the provider questionnaires and focus groups will be subject to three broad phases of analysis [57]. First, in the preparation phase, the open questionnaire response data and focus group transcriptions will be read and re-read in order to obtain a sense of the entire dataset. Second, the data will be organised, with initial open coding and categorisation of the data subsequently grouped into more refined categories and subcategories. In the final phase, the analysing process and results will be reported, through the presentation of emergent categories, and an overarching narrative. Two separate researchers will conduct the coding and categorisation of all data, with any key areas of divergence/discrepancy discussed and resolved with a third researcher. Data integration will occur primarily at the development stage, whereby themes identified in the structured questionnaire data will be used to inform the discussion topics of the provider focus groups [58].

## Discussion

Despite the fact that there are several promising implementation strategies available to policymakers and practitioners at present, uptake of ASBI remains limited and inconsistent in most countries worldwide. Moreover, we still lack a sound understanding what works, in which context, when and specifically for whom when it comes to tackling drinking in general populations.

This is the first trial to assess the feasibility of conducting a fully randomised controlled trial of the effectiveness of ASBI in primary care in Kazakhstan. By employing the pragmatic yet robust RE-AIM framework to evaluate implementation, the trial aims to deliver a comprehensive assessment of the impact of the intervention packages on the target population. Further, by exploring the feasibility and acceptability of implementing ASBI from both patients’ and physicians’ perspectives, the study also aims to provide an in-depth understanding of the processes shaping translation of ASBI into this specific setting. In doing so, the study will provide valuable new insights into the potential effectiveness of ASBI in routine PHCU practice, and an improved understanding of which factors and processes influence the effective (and thus sustainable) implementation of such interventions across Kazakhstan.

## Data Availability

No datasets were used or analysed in drafting this protocol.

## Abbreviations

ASBI: Alcohol screening and brief intervention
AUDIT: Alcohol Use Disorders Identification Test
AUDIT-C: Alcohol Use Disorders Identification Test-Consumption
CRF: Case-report form
GCP: Good Clinical Practice
ICC: Intra-class coefficient
ICH: International Conference on Harmonisation
IEC: Independent Ethics Committee
PHC: Primary health care
PHCU: Primary health care unit
RE-AIM: Reach-Effectiveness-Adoption-Implementation-Maintenance Framework
SAAPPQ: Shortened Alcohol and Alcohol Problems Perception Questionnaire
WHO: World Health Organization

## References

[1] GBD 2016 Alcohol Collaborators. Alcohol use and burden for 195 countries and territories, 1990–2016: a systematic analysis for the Global Burden of Disease Study 2016. Lancet. 2018. 10.1016/S0140-6736(18)31310-2 PMC6148333 30146330

[2] Rehm J, Mathers C, Popova S, Thavorncharoensap M, Teerawattananon Y, Patra J (2009). Global burden of disease and injury and economic cost attributable to alcohol use and alcohol-use disorders. Lancet..

[3] Rehm J, Gmel GE, Gmel G, Hasan OSM, Imtiaz S, Popova S (2017). The relationship between different dimensions of alcohol use and the burden of disease-an update. Addiction (Abingdon, England).

[4] Holmes MV, Dale CE, Zuccolo L, Silverwood RJ, Guo Y, Ye Z (2014). Association between alcohol and cardiovascular disease: Mendelian randomisation analysis based on individual participant data. BMJ (Clinical research ed).

[5] Knott CS, Coombs N, Stamatakis E, Biddulph JP (2015). All cause mortality and the case for age specific alcohol consumption guidelines: pooled analyses of up to 10 population based cohorts. BMJ (Clinical research ed).

[6] Di Castelnuovo A, Costanzo S, Bagnardi V, Donati MB, Iacoviello L, de Gaetano G (2006). Alcohol dosing and total mortality in men and women: an updated meta-analysis of 34 prospective studies. Arch Intern Med..

[7] Karriker-Jaffe KJ, Lonn SL, Cook WK, Kendler KS, Sundquist K (2018). Chains of risk for alcohol use disorder: mediators of exposure to neighborhood deprivation in early and middle childhood. Health Place..

[8] Carrell SE, Hoekstra M, West JE. Does drinking impair college performance? Evidence from a Regression Discontinuity Approach: National Bureau of Economic Research Working Paper Series; 2010. p. 16330.

[9] Wolaver AM (2007). Does drinking affect grades more for women? Gender differences in the effects of heavy episodic drinking in college. Am Econ.

[10] Laslett AM, Room R, Dietze P, Ferris J (2012). Alcohol’s involvement in recurrent child abuse and neglect cases. Addiction (Abingdon, England).

[11] Navarro HJ, Doran CM, Shakeshaft AP (2011). Measuring costs of alcohol harm to others: a review of the literature. Drug Alcohol Depend.

[12] Taylor B, Rehm J (2012). The relationship between alcohol consumption and fatal motor vehicle injury: high risk at low alcohol levels. Alcohol Clin Exp Res.

[13] Taylor B, Irving HM, Kanteres F, Room R, Borges G, Cherpitel C (2010). The more you drink, the harder you fall: a systematic review and meta-analysis of how acute alcohol consumption and injury or collision risk increase together. Drug Alcohol Depend.

[14] Standerwick K, Davies C, Tucker L, Sheron N (2007). Binge drinking, sexual behaviour and sexually transmitted infection in the UK. Int J STD AIDS.

[15] Aicken CR, Nardone A, Mercer CH (2011). Alcohol misuse, sexual risk behaviour and adverse sexual health outcomes: evidence from Britain's national probability sexual behaviour surveys. J Public Health (Oxf).

[16] World Health Organization (2013). World health report 2013: research for universal health coverage.

[17] World Health Organization (2014). Global status report on alcohol and health 2014.

[18] World Health Organisation. Global Health Observatory data repository. Alcohol - Data by country. Geneva: WHO; 2018 [Available from: http://apps.who.int/gho/data/view.main.1800?lang=en.

[19] Republican Center for Health Development. The health of the population of the Republic of Kazakhstan and the activities of health organizations in 2010. Statistical collection; Astana-Almaty, 2011 [Available from: http://www.rcrz.kz/files/sbornik/%D0%A1%D0%B1%D0%BE%D1%80%D0%BD%D0%B8%D0%BA%202010-2011.pdf.

[20] World Health Organisation (2010). Global Health Observatory (GHO) data: Kazakhstan.

[21] Republican Center for Health Development. The health of the population of the Republic of Kazakhstan and the activities of health organizations in 2017. Statistical collection; Astana-Almaty, 2018 [Available from: http://www.rcrz.kz/files/sbornik/sbornik_2018-converted.pdf.

[22] Ministry of Health Care of the Republic of Kazakhstan. Analytical Material of The Extended Collegium of the Ministry of Healthcare of The Republic of Kazakhstan. Astana: 2017 [Available from: http://www.rcrz.kz/old/docs/broshura.pdf.

[23] Lock C, Wilson G, Kaner E, Cassidy P, Christie M, Heather N (2009). A survey of general practitioners’ knowledge, attitudes and practices regarding the prevention and management of alcohol-related problems: an update of a World Health Organisation survey ten years on.

[24] Anderson P, O’Donnell A, Kaner E (2017). Managing alcohol use disorder in primary health care. Curr Psychiatry Rep.

[25] Bernstein E, Topp D, Shaw E, Girard C, Pressman K, Woolcock E (2009). A preliminary report of knowledge translation: lessons from taking screening and brief intervention techniques from the research setting into regional systems of care. Acad Emerg Med..

[26] Lock CA, Kaner EF (2004). Implementation of brief alcohol interventions by nurses in primary care: do non-clinical factors influence practice?. Fam Prac.

[27] Rubinsky AD, Dawson DA, Williams EC, Kivlahan DR, Bradley KA (2013). AUDIT-C scores as a scaled marker of mean daily drinking, alcohol use disorder severity, and probability of alcohol dependence in a U.S. general population sample of drinkers. Alcohol Clin Exp Res.

[28] Kriston L (2008). Meta-analysis: are 3 questions enough to detect unhealthy alcohol use?. Ann Intern Med.

[29] Reducing Alcohol Harm: Health services in England for alcohol misuse - National Audit Office (NAO) Report. 2008.

[30] Miller WR, Benefield RG, Tonigan JS (1993). Enhancing motivation for change in problem drinking: a controlled comparison of two therapist styles. J Consult Clin Psychol..

[31] Kaner EF, Beyer FR, Muirhead C, Campbell F, Pienaar ED, Bertholet N (2018). Effectiveness of brief alcohol interventions in primary care populations. Cochrane Database Syst Rev.

[32] Kaner EF, Beyer F, Dickinson HO, Pienaar E, Campbell F, Schlesinger C (2007). Effectiveness of brief alcohol interventions in primary care populations. Cochrane Database Syst Rev..

[33] Bray JW, Zarkin GA, Davis KL, Mitra D, Higgins-Biddle JC, Babor TF (2007). The effect of screening and brief intervention for risky drinking on health care utilization in managed care organizations. Med Care..

[34] Cuijpers P, Riper H, Lemmers L (2004). The effects on mortality of brief interventions for problem drinking: a meta-analysis. Addiction.

[35] Babor T, Higgins-Biddle J (2001). Brief Intervenation for hazardous and harmful drinking: a manual for use in primary care.

[36] NICE (2010). Alcohol-use disorders: preventing the development of hazardous and harmful drinking: NICE public health guidance 24.

[37] Swedish National Institute of Public Health (2010). Alcohol issues in daily healthcare. The Risk Drinking Project - background strategy and results.

[38] McCormick R, Docherty B, Segura L, Colom J, Gual A, Cassidy P (2010). The research translation problem: alcohol screening and brief intervention in primary care—real world evidence supports theory. Drugs Educ Prevent Policy..

[39] Moyer VA, on behalf of the USPSTF (2013). Screening and behavioral counseling interventions in primary care to reduce alcohol misuse: U.S. preventive services task force recommendation statement. Ann Intern Med..

[40] van Beurden I, Anderson P, Akkermans RP, Grol RP, Wensing M, Laurant MG (2012). Involvement of general practitioners in managing alcohol problems: a randomized controlled trial of a tailored improvement programme. Addiction..

[41] Seppanen KK, Aalto M, Seppa K (2012). Institutionalization of brief alcohol intervention in primary health care-the Finnish case. Alcohol Clin Exp Res..

[42] Brown J, West R, Angus C, Beard E, Brennan A, Drummond C (2016). Comparison of brief interventions in primary care on smoking and excessive alcohol consumption: a population survey in England. Br J Gen Pract..

[43] Joseph J, Basu D (2017). Efficacy of brief interventions in reducing hazardous or harmful alcohol use in middle-income countries: systematic review of randomized controlled trials. Alcohol Alcohol.

[44] L'engle KL, Mwarogo P, Kingola N, Sinkele W, Weiner DH (2014). A randomized controlled trial of a brief intervention to reduce alcohol use among female sex workers in Mombasa, Kenya. J Acquir Immune Defic Syndr.

[45] Mertens JR, Ward CL, Bresick GF, Broder T, Weisner CM (2014). Effectiveness of nurse-practitioner-delivered brief motivational intervention for young adult alcohol and drug use in primary care in South Africa: a randomized clinical trial. Alcohol Alcohol.

[46] Anderson P, Laurant M, Kaner E, Wensing M, Grol R (2004). Engaging general practitioners in the management of hazardous and harmful alcohol consumption: results of a meta-analysis. J Stud Alcohol.

[47] Funk M, Wutzke S, Kaner E, Anderson P, Pas L, McCormick R (2005). A multicountry controlled trial of strategies to promote dissemination and implementation of brief alcohol intervention in primary health care: findings of a World Health Organization collaborative study. J Stud Alcohol.

[48] Bush K, Kivlahan DR, McDonell MB, Fihn SD, Bradley KA (1998). The AUDIT alcohol consumption questions (AUDIT-C): an effective brief screening test for problem drinking. Ambulatory Care Quality Improvement Project (ACQUIP). Alcohol Use Disorders Identification Test. Arch Intern Med..

[49] Saunders JB, Aasland OG, Babor TF, de la Fuente JR, Grant M (1993). Development of the Alcohol Use Disorders Identification Test (AUDIT): WHO Collaborative Project on Early Detection of Persons with Harmful Alcohol Consumption II. Addiction.

[50] Hester RK, Miller WR (2003). Handbook of alcoholism treatment approaches. Effective alternatives.

[51] World Health Organisation. WHO alcohol brief intervention training manual for primary care Copenhagen: World Health Organisation; 2017 [Available from: http://www.euro.who.int/ru/health-topics/disease-prevention/alcohol-use/publications/2017/who-alcohol-brief-intervention-training-manual-for-primary-care-2017.

[52] Anderson P, Coulton S, Kaner E, Bendtsen P, Kłoda K, Reynolds J (2017). Delivery of brief interventions for heavy drinking in primary care: outcomes of the ODHIN 5-Country Cluster Randomized Trial. Ann Fam Med.

[53] Anderson P (1985). Managing alcohol problems in general practice. Br Med J.

[54] Glasgow R E, Vogt T M, Boles S M (1999). Evaluating the public health impact of health promotion interventions: the RE-AIM framework. American Journal of Public Health.

[55] Republican Scientific and Practical Center on Medical and Social Problems of Drug Addiction (2014). The Report on implementation UNODC Global project “TREATNET II” in the Republic of Kazakhstan (2009-2014).

[56] Pope C, Ziebland S, Mays N, Pope C, Mays N (2006). Analysing qualitative data. Qualitative Research in Health Care. 3.

[57] Elo S, Kyngas H (2008). The qualitative content analysis process. Journal of advanced nursing..

[58] Teddlie C, Tashakkori A (2006). A general typology of research designs featuring mixed method. Res Schools..

[59] International Committee of Medical Journal Editors. Recommendations for the Conduct, Reporting, Editing, and Publication of Scholarly Work in Medical Journals. 2018 [Available from: http://www.icmje.org/icmje-recommendations.pdf. 25558501

[60] Chan AW, Tetzlaff JM, Altman DG, Laupacis A, Gotzsche PC, Krleza-Jeric K (2013). SPIRIT 2013 statement: defining standard protocol items for clinical trials. Ann Intern Med..

